# Indents

**Published:** 1903-07-15

**Authors:** 


### INDENTS.

**☞ Brief Articles of Technical or Scientific Value will receive notice and comment. Contributions invited.**

**To Stop Hemorrhage After Extracting.** Dissolve antipyrene in campho-phenique, until a saturated solution is obtained. Make a plug of cotton to conform to socket. Saturate this with the solution, and pack into socket; apply compress if necessary. I have had good results with this when everything else failed.—P. S. Turner, in *Texas Journal.*

**A Point About Ether Anesthesia.** A point of great importance in ether anesthesia is to remember that after its administration the patient has all his blood vessels dilated and has lost heat largely, so that the administration, especially if at all prolonged, should take place in a warmed room, and every precaution against catching cold should be taken.—B. W. Hogarth, *British Journal.*

**Cleaning Glass Cement-Slabs.** Every practitioner knows how difficult it is to remove the cement which adheres to a glass cement slab. Usually a knife is resorted to and the slab presents a scratched appearance therefrom. I accidentally discovered that dilute nitric acid will remove all cement particles, no matter how hard, and the slab, after being rinsed in water and dried, will have a clean, smooth surface.—Geo. Zederbaum, *Dental Review.*

**A Hint in Root-Fiiling.** Here is a little "trick" that occasionally serves me in conservative treatment of the pulp—new to me, this, but so very simple that it is most likely, very old.

With the dressing and cap in position, mix a bit of cement to the consistence of thick cream and place a drop upon the cap. A few quick, vigorous, and properly directed puffs from the air syringe will spread the cement nicely over the cap and dressing, and without exerting pressure. When set, this serves to hold the cap in place while the filling proper is inserted.—L. S. Edwards, in *Cosmos*.

**Carborundum for Painless Work.**

To make the heart glad, get a couple of porte-polishers, No. 305 of combination catalogue, and a box of corborundum points (cylinders, not rubber). These points may be set in the mandrel with modeling compound. A little hole drilled in the side to expel the air is still better, and offers a vent for the removal of a broken point. These points are just plain cylinders, but one mandrel point should be sharpened on a revolving wheel for opening up small cavities. Sensitive patients and children will let you work with this when they would not put up with burs for a moment.—W. H. Albright. in *Cosmos*.

**To prevent Melotte's Metal from Becoming Brittle.**

If whiting be used as a separating material in making dies and counter-dies with Melotte's metal, it should be thoroughly removed before the metal is remelted. The heat in fusing breaks up the carbonate radical of the whiting, and lime (calcium oxid), is formed, which, if incorporated in the alloy, makes it brittle. The lime can be chemically and easily removed by dipping the ingot in dilute hydrochlorid acid (ten percent). This will convert the lime into soluble calcium chlorid and the soluble salt can be thoroughly washed off with water.—J. P. Buckley, in *Review*.

**Anchoring Lower Plates.**

I would not allow the best dentist in the world to take out my last lower tooth, if I were going to wear a lower plate, unless it was wabbling. If there was a tooth in the mouth that had a permanent attachment to the jaw I would save it and anchor to it.

You know that you can hold a fractious horse with a piece of string tied to a post. This will steady him. The same thing applies to anchoring a plate in the lower jaw. When it is anchored a patient will not play with it, because it is not annoying. On the other hand, if you do not anchor it, the patient will invariably keep fussing with it.—W. H. Taggart, in *Review*.

**Hydronaphthol in Cavity Linings.** There are many cases of fillings that are not very close to the pulp and where the pulp died, not because of thermal changes, but because of micro-organisms remaining in the dentin. And I believe from my own short experience that a mild and constant antiseptic of some kind mixed with the cement will obviate the death of pulps in such cases. It is my custom, and has been for ten or twelve years, to line the bottoms of all deep cavities, whether they are encroaching close upon the pulp or not. I always incorporate into that lining hydro-naphthol. I think possibly that Dr. Harlan's suggestion of odorless and colorless beta-naphthol is an improvement over the old.—E. R. Carpenter, in *Review*.

**Restoring a Broken Incisor.** I bevelled the tooth linguo-labially slightly, then made an open-faced gold crown of proper length and slipped it on the tooth, and took an impression with modelling compound, made a model with a mixture of plaster and pumice with open-faced crown in position. I then took a porcelain facing of suitable size and color to finish out required length of incisor, backed it with gold plate, ground it to fit the bevelled incisor, making as near a perfect joint as possible. The natural tooth was thicker than facing, but I cut grooves in lingual portion of open-faced crown, through which the pins of the facing protruded when facing was in position; invested and soldered as I would a Richmond crown; finished and cemented into place. The joint is perceptible, but the gentleman is the possessor of a heavy mustache and it is hid from view. It has been in position for fifteen or eighteen months and is a success thus far.—C. W. St. Clair, in *Dental Brief*.

**Odors in Dental Office.** The odor of medicines used in the operating room should not be allowed to permeate the office. It usually arises from the waste cotton saturated in the medicine and left lying on the floor or in a waste-paper basket, and not from the bottles containing the medicine if they are kept securely corked. The odor may not be noticed by the dentist, as he is constantly in the presence of it, but his patients immediately detect it as soon as they enter the office, and its effect usually creates a fear of the operation about to be performed. A suitable receptacle should be kept at hand, into which the waste cotton can be placed immediately after using, and the cotton can be destroyed before the next patient takes the chair. Where practical the window might be opened for a moment. The office should be well ventilated at all times.—*Review*.

**Poisoning With Suprarenal Solution.** The essayist calls attention to a case of injection of suprarenal solution which was followed by toxic phenomena. The injection was performed in order to arrest hemorrhage incident to urethrotomy. The hemorrhage was arrested immediately, but the patient fainted and suffered from convulsions that lasted for several hours. Dr. Von Furth calls attention to the fact that intravenous injections of small doses of suprarenal solution in animals have caused toxic phenomena, and states that subcutaneous injections are likewise followed by these toxic and dangerous symptoms. He therefore says that the use of suprarenal preparations in dentistry should certainly be carried out with caution until further experiments shall have determined the exact *modus operandi* to be followid in cases of this nature. He also refers to other cases of poisoning caused by the use in various ways of suprarenal preparations.—*Cosmos*.

**Platinum Solder.** Pure gold for solder, if you are using a high-fusing porcelain, is entirely out of place; I do not think it should ever be used. The one main point, one of the finest things in the development of porcelain work, is in the use of solder that will not be fused at the highest point to which you carry your heat for fusing your porcelain. Use platinum solder, twenty-five to forty percent. If you use the highest-fusing porcelain you can use forty percent solder. Now, in order to use this platinum solder, it is not necessary to have special appliances. If you have a common nitrous cylinder and a yoke, and will attach a rubber tube to it, all you need is an ordinary blow-pipe. Attach your blow-pipe to the nitrous oxid cylinder and the illuminating gas. You will find that you get a flame that will melt the forty-percent solder just as easy as would a special make of blow-pipe.—Dr. Baker, in *Cosmos*.

**Statistics of Ethyl Chlorid Anesthesia.** In the last four years or so Ethyl Chlorid —which was first used under a misapprehension by Lotheisen, of Innsbruck—has to a large extent replaced Ethyl Bromid. The ordinary pure Ethyl Chlorid, $C_2H_5C_1$, sometimes called by the proprietary names of Kelene, or Narcotile, is the drug used. Various forms of mask have been recommended. The earlier ones, such as Breuer's, allowed of admixture of the vapor with air; more recently, in order to avoid the excitement sometimes caused by the mixture, air has been largely excluded until narcosis is complete. In my last three hundred cases I have used Ormsby's ether inhaler, which quite fulfills this object. I use the tubes of 60 gm., graduated, and emitting a larger spray than usual. Anesthesia occurs very rapidly usually, in from one-half to one minute—that is, more quickly than is the case with nitrous oxid. Excitement is absent. During narcosis the color is improved; the pulse, so far as I can ascertain, is about normal in quality and the respiration stimulated,. In deep anesthesia stertor is usually present. The awakening is very rapid and complete.

Seitz, of Constance, has recently collected 16,000 cases, with one death, that of a very bad subject.—W. J. McCardie, *Treatment.*

**Alopecia Areata of the Mustache of Dental Origin.** A man, aged thirty-five years, who had suffered much from carious teeth, of which he had a great number, presented a patch of alopecia areata in the mustache over the upper-right lateral incisor. This tooth, after a severe neuralgia of both jaws had remained very sensitive, and the patch of alopecia had developed over it at the same time vesicles identical in appearance with those of herpes had appeared on the corresponding part of the gum. The hairs about the borders of the patch were easily extracted, and presented the usual appearances observed in this disease. The skin was thinned and presented marked anesthesia. There were no other patches of alopecia. The patient was directed to have the mouth cleansed and the teeth looked after by a dentist, and was given quinin internally in moderate doses. Shortly after the jaws had been put in good condition, the sensitive incisor having been extracted, the patch of alopecia ceased to extend. Later, the neuralgia ceased, the sensibility of the skin returned, and at the end of about four months the plaque was completely covered with hair.—*Amer. Jour. of the Med. Sciences.*

**A Method of Pulp Devitalization.** I have had such marked success in my method of devitalizing pulps of teeth during the last two years that I feel justified in describing it.

First, apply a saturated solution of carbolic acid and cocain (after cleaning the cavity). Leave it in a minute, so as to get the good effects of the cocain. Then apply the devitalizing paste (directly against the pulp if there be an exposure), and seal in with oxyphosphate of zinc. I *always* use the cement for the *first* application and think this a large factor in the painlessness of the treatment, for if put in while soft it will protect the pulp from pressure. To make the removal of the cement easy, I dilute the phosphoric acid fifty percent or more with water before adding the powder. This necessitates placing it in the cavity quickly while it is soft, as it hardens more rapidly than with the undiluted liquid.

I can say that I have had but very few cases where much pain has been produced, the almost universal result being either absolutely painless or only a dull pain for an hour or two. There have been a few exceptions, where there was marked pain, and I think in every case it was where there was an unusual formation of secondary dentin.—William N. Bush, in *Dental Brief*.

**Purifying and Preserving Sheet Wax.** A good formula for sheet wax is one pound of beeswax and half a pound of paraffine, melted together and thoroughly mixed. To renovate the wax after it has been used, put three quarts of water in a pot, heat to the boiling point, add to it one ounce of oxalic acid and immediately throw in the waste wax. After it is melted allow it to boil for five or ten minutes, then set it in a place where it will cool slowly. When it is hard the impurities can be scraped from the under surface. It is then ready to be made into sheets. To prevent the wax from being discolored by overheating it should be melted in a double boiler. Make three or four receiving trays of common tin-plate such as tinsmiths use, about seven inches square, with a raised edge about one-quarter of an inch high. Amalgamate the surface of these trays with mercury to prevent the wax from sticking to them. Set the trays on a level surface and pour the melted wax into them quickly. With a little experience the sheets can be made just the proper thickness for use. When the wax becomes set, chill it in cold water and it is easily removed from the trays. Cut each sheet into four parts, and each part will wax up one model.—Dr. W. A. Brownlee, *Dominion Journal*.

**What Should be Taught in the Fourth Year.** We do not want to add a fourth year to our dental course just to put on an extra frill or two. We must have a four-year's course, because we cannot do all the work in three years that ought to be done. Then we must have a hospital course or a laboratory course in special investigation work, or something of that kind. Both these things are important. Gentlemen, the greatest need of the dental profession to-day is to make professional men instead of artisans. We are in greater danger than most men realize of becoming a company of skilled workmen instead of a great profession.

Previous to twenty or thirty years ago we had a few professional men in our ranks, and we had a great many mechanics and artisans, and if we are not careful the time will come when we will have the same thing again, and it will be the fault of the colleges if the rank and file of our practitioners are merely skilled mechanics and business men instead of being professional men. We shall not be without professional men, because many will become such by force of personal character, ambition and attainments. But the purpose of dental education ought to be to lift up the whole number of graduates into professionalism. If you are going to do that you must make students as well as operators and mechanical men. In order to do that you must teach them something of those sciences which are at the foundation of all professional knowledge and practice. You must awaken in the students some interest in these subjects; give them time enough to learn something about them, and the four-year's course should be used more for the purposes of strengthening this aspect of dental education than for anything else.

I do not wish to disparage for a moment clinical or technical instruction; in fact, it should be made stronger rather than weaker, but the weak side of dental education to-day is the scientific and the professional side.—Dr. E. Noyes, in *Dental Review.*

